# Public perceptions and influencing factors of seasonal influenza vaccine uptake in Makkah region, Saudi Arabia: a cross-sectional study

**DOI:** 10.3389/fpubh.2025.1534176

**Published:** 2025-02-18

**Authors:** Mohammed S. Alharthi, Abdullah A. Alshehri, Fahad H. Baali, Nawaf Awd Altuwairqi, Meshari Hassan Altalhi, Rayan Azib Almalki, Meshal Khalid Aljuaid, Majed A. Algarni, Mohmmed S. Alzahrani, Nasser M. Alorfi, Wadia S. Alruqayb

**Affiliations:** ^1^Department of Clinical Pharmacy, College of Pharmacy, Taif University, Taif, Saudi Arabia; ^2^Department of Pharmacology and Toxicology, College of Pharmacy, Umm Al-Qura University, Makkah, Saudi Arabia

**Keywords:** seasonal influenza vaccine, influenza, hesitancy, public, Makkah region, Saudi Arabia

## Abstract

**Background:**

Seasonal influenza vaccination is essential for reducing the risk and impact of influenza. Makkah region in Saudi Arabia, a destination for millions of pilgrims during Hajj and Umrah, presents a unique context for this study. Therefore, this research investigates the prevalence and influencing factors of influenza vaccine uptake among the public in Makkah region, Saudi Arabia.

**Method:**

This cross-sectional study was conducted in Saudi Arabia between February and June 2024. The main researcher developed the questionnaire, which was reviewed by five academics and then piloted with 20 individuals for validation. An online questionnaire was used, targeting residents aged 18 and over with internet access. A snowball sampling method was applied. Data were collected via Google Forms and analyzed using SPSS version 26, employing descriptive statistics such as frequencies, percentages, and means.

**Results:**

The total number of respondents is 450 participants, with a response rate of 4.5%. Most respondents were male (71.1%) and aged between 18 and 30 years (44.5%). Education levels varied, with 57.5% holding a bachelor’s degree. Vaccination uptake showed 65.1% had received the vaccine, with 31.1% confident it is safe. Despite this, 58.8% did not get vaccinated this season. Confidence in the vaccine’s effectiveness was 59.5%, though 41.2% reported breakthrough infections. Accessibility was generally rated easy (57.7%). Social pressure (17.4%), trust in health institutions (36.9%), and COVID-19 concerns (36.7%) significantly influenced vaccination decisions. The likelihood of vaccination next season displayed polarized views.

**Conclusion:**

This study provides significant insights about vaccine hesitancy that may inform future research endeavors and practical applications. Understanding the various factors that impact the adoption of influenza vaccines offers valuable insights for developing targeted interventions and public health policies to improve vaccination rates. This study enhances theoretical understanding and practical strategies to encourage influenza vaccination, thereby improving public health initiatives in the area and beyond.

## Introduction

Seasonal influenza (flu) is a significant global public health concern, contributing to annual morbidity and mortality (1). Each year, particularly during the winter, seasonal influenza affects up to one billion individuals worldwide, with most cases being mild. However, the World Health Organization (WHO) estimates that 3 to 5 million cases develop into severe illness, leading to 290,000–650,000 respiratory deaths annually (2). The burden of influenza extends beyond individual health, imposing economic and healthcare system challenges worldwide. In Saudi Arabia, the incidence of influenza-like illnesses (ILIs) and severe acute respiratory infections (SARIs) has increased significantly, with a notable spike in 2022 compared to previous years (3). The epidemiology of influenza in the Middle East and North Africa shows that influenza A and B account for 76.5 and 23.5% of cases, respectively, with influenza A being dominant in 86.8% of seasons (4). While most countries exhibit seasonality patterns similar to the Northern Hemisphere, regions such as the Arabian Peninsula experience secondary peaks, mainly due to large-scale population movements. Despite the availability of influenza vaccines, uptake remains suboptimal globally. A 2021 study examining demographic and educational influences on influenza vaccine awareness in Saudi Arabia found that 50% of surveyed adults were vaccinated. Meanwhile, 42% exhibited vaccine hesitancy due to a lack of awareness and safety concerns (5). Vaccination is widely recognized as an effective preventive measure, reducing hospitalization and mortality rates (6, 7). However, vaccination coverage varies significantly across geographic locations and demographic groups, with disparities influenced by socioeconomic status, cultural beliefs, and misinformation (8, 9). Religious and cultural perceptions further impact vaccine uptake, with some individuals considering vaccinations unnecessary or unnatural interventions (10). A global study on vaccine hesitancy identified concerns about side effects, perceived low risk of infection, and distrust in vaccine manufacturers as primary barriers to vaccine acceptance (11). In Saudi Arabia, factors such as public trust in health institutions, accessibility, and social influence have significantly affected vaccination decisions (12). Makkah presents a unique epidemiological setting, as it hosts millions of international visitors annually for Hajj and Umrah, creating an ideal environment for the rapid transmission of respiratory illnesses. Given this high-risk context, ensuring adequate influenza vaccine coverage among residents and visitors is a public health priority (13). While previous research has explored vaccine acceptance in diverse global populations, there remains a notable gap in understanding the attitudes and behaviors of individuals in Makkah regarding seasonal influenza vaccination. This cross-sectional study aims to address this gap by investigating public perceptions, uptake rates, and key factors influencing influenza vaccination in Makkah. By identifying barriers and facilitators to vaccine uptake, the findings will contribute to developing targeted public health interventions aimed at improving vaccination coverage and reducing the burden of seasonal influenza in this high-density region.

## Methods

### Study setting

The study employed a cross-sectional design targeting the adult population in Makkah region of Saudi Arabia. An online questionnaire was distributed among the population. It was conducted between February 2024 and June 2024. The inclusion criteria involved males and females 18 years old and over and participants with internet access who could participate in this study. The exclusion criteria were individuals outside Makkah region, those under 18 years old, and those without internet access.

### Questionnaire development, validity and reliability

The main researcher (MA) developed the questionnaire based on the study’s aim and objectives after reviewing the literature of similar studies (14–17). Five academics critically assessed the questionnaire for accuracy, relevance, and appropriateness. The questionnaire consists of five parts with 16 closed-ended questions. These include demographics, vaccine knowledge, attitudes, vaccination practices, and factors influencing vaccine uptake. The questions cover the respondent’s demographics, knowledge about the influenza vaccine, attitudes toward its safety and effectiveness, personal vaccination practices, and factors influencing their decision to get vaccinated. The questionnaire included items assessing participants’ knowledge about the influenza vaccine, covering aspects such as its purpose, effectiveness, and recommended groups for vaccination. These items were designed to provide insight into respondents’ awareness levels. The findings related to knowledge are presented in the results section. Additionally, reliability analysis demonstrated a Cronbach’s Alpha of 0.766, reflecting an acceptable level of internal consistency. When computed based on standardized items, Cronbach’s Alpha slightly adjusted to 0.761, indicating a stable and consistent measurement scale.

### Sampling and sample size

The study adopted a snowballing sampling technique to maximize the number of participants. The sample size was determined using the Cochrane sample size formula: *n = Z^2^(1 − p)/d^2^*. Here, *n* is the sample size, *Z* is the critical value for a 95% confidence interval, *p* is the anticipated proportion (50%), and *d* is the margin of error (set at 0.05). The minimum sample size was 384, but the researchers targeted 450 respondents to account for potential non-response.

### Response rate calculation

The response rate was estimated by dividing the number of completed responses by the approximate number of individuals who received or had access to the survey link. Since the survey was distributed via multiple online platforms, including social media and direct messaging, the denominator was approximated based on platform reach metrics and engagement data. The response rate was calculated as: *(Response Rate = (Total Completed Responses / Estimated Recipients) × 100)*. Although estimating the exact number of recipients is challenging, this method reasonably approximates participation.

### Piloting the questionnaire

To ensure validity, the questionnaire was piloted with 20 individuals from diverse demographics, such as age and education level. The piloted results were not included in the final analysis. The questionnaire was distributed via social media platforms like X, WhatsApp, and Telegram.

### Data collection and analysis

The data was collected through an online questionnaire using Google Forms (which is compliant with the General Data Protection Regulation (GDPR)). They were entered into Statistical Package for the Social Sciences (SPSS) version 26 to analyze the basic features of the collected data. Descriptive analysis, including frequencies with percentages and means, was performed. Consent was obtained at the beginning of the questionnaire. In addition to descriptive statistics, Pearson correlation analysis was performed to examine the relationships between the likelihood of receiving the flu vaccine and influencing factors. The correlation coefficients (*r*) and corresponding *p*-values were used to assess the strength and significance of the relationships between variables. The factors analyzed included trust in vaccine safety, trust in efficacy, accessibility, peer pressure, trust in information, and awareness. These analyses aimed to identify key influencing factors associated with flu vaccine uptake within the study population.

### Ethical considerations

Ethical approval was obtained from the Taif University Ethical Committee (application number 45–283). Key ethical considerations included confidentiality and informed consent. Participants were informed about the study’s purpose, procedures, risks, and benefits. Researchers ensured participants’ privacy by protecting their identity and sensitive information.

## Results

### Demographic characteristics of the respondent

The total number of respondents is 450, with a response rate of 4.5%. Table 1 shows the demographic characteristics of the respondents, which indicate a higher proportion of males (71.1%) than females (28.9%). The age distribution shows that the largest group is aged 18–30 years (44.5%), followed by those aged 41–50 years (19.9%), 51–60 years (19.7%), and 31–40 years (12.3%). Educational attainment among the respondents is primarily at the bachelor’s degree level (57.5%), with 12.3% possessing postgraduate qualifications, 22.4% having completed high school or an equivalent, and 7.8% having less than a high school education. Employment status reveals that 47.2% of the respondents were employed, 32.9% were students, 12.3% were retired, and 7.4% were unemployed.

**Table 1:** Characteristics of questionnaire respondents.

Category	Characteristics	*n* (%)
Gender	Male	318 (71.1)
	Female	129 (28.9)
Age (years)	18–30	199 (44.5)
	31–40	55 (12.3)
	41–50	89 (19.9)
	51–60	88 (19.7)
Education	Less than high school	35 (7.8)
	High school or equivalent	100 (22.4)
	Bachelor’s degree	257 (57.5)
	Postgraduate	55 (12.3)
Employment status	Student	147 (32.9)
	Employee	211 (47.2)
	Unemployed	33 (7.4)
	Retired	55 (12.3)

### Response rate

A total of 450 participants completed the questionnaire, yielding a response rate of 4.5%. While the survey was widely distributed online, participation remained limited, reflecting possible challenges in engagement and outreach effectiveness.

### Pilot study findings

A pilot study was conducted with 20 participants to evaluate the questionnaire’s clarity, comprehensibility, and reliability before administering it to the main study population. The participants included 12 males (60%) and eight females (40%) aged 18 to 60. The majority (55%) were aged between 18 and 30 years. Educational backgrounds varied, with 50% holding a bachelor’s degree, 30% having completed high school, and 20% possessing postgraduate qualifications. Regarding employment status, 45% were employed, 35% were students, and 20% were unemployed. The participants provided valuable feedback regarding the wording and structure of the questionnaire. Minor adjustments were made to three questions to enhance clarity and eliminate ambiguity, ensuring a better understanding of the survey items. The pilot study results were not included in the final data analysis but were instrumental in refining the questionnaire to enhance its validity and reliability. No significant issues were reported, confirming the appropriateness of the questionnaire for the main study.

### Participants’ knowledge of seasonal influenza vaccine

Table 2 shows that participants’ knowledge of the seasonal influenza vaccine varies. 62.5% (*n* = 281) correctly identified that the vaccine is recommended annually for high-risk populations, including older adults, pregnant women, and individuals with chronic diseases. However, 27.4% (*n* = 123) were unsure about the recommended frequency, and 10.1% (*n* = 46) incorrectly believed that one dose in a lifetime is sufficient. Regarding vaccine effectiveness, 64.7% (*n* = 291) correctly stated that the vaccine reduces, but does not entirely prevent, influenza infections. In contrast, 18.2% (*n* = 82) believed the vaccine completely prevents flu, while 17.1% (*n* = 77) were uncertain. Additionally, 55.8% (*n* = 251) correctly recognized that the vaccine does not cause influenza, but 28.5% (*n* = 128) mistakenly believed that receiving the vaccine could lead to flu infection, highlighting a common misconception.

**Table 2:** Participants’ knowledge of seasonal influenza vaccine.

Question	Correct response	*n* (%)	Incorrect response	*n* (%)	Unsure *n* (%)
The influenza vaccine should be taken annually by high-risk individuals.	Yes	281 (62.5)	No	46 (10.1)	123 (27.4)
The influenza vaccine completely prevents flu.	No	291 (64.7)	Yes	82 (18.2)	77 (17.1)
The influenza vaccine can cause influenza.	No	251 (55.8)	Yes	128 (28.5)	71 (15.7)

### Attitudes and practices toward seasonal influenza vaccination

Table 3 illustrates respondents’ attitudes toward the seasonal influenza vaccine and their vaccination practices. Most participants (65.1%) reported receiving the vaccine at least once, with 19.5% receiving it occasionally and 18.8% annually. However, a substantial proportion (58.8%) did not receive the influenza vaccine during the most recent season, indicating potential barriers to uptake despite largely positive attitudes toward vaccine safety and efficacy. Confidence in vaccine safety was generally high, with 31.1% being very confident and 24.2% somewhat confident, while 28.9% remained neutral. A smaller percentage expressed concerns about vaccine safety, with 11.2% somewhat concerned and 4.7% very concerned. These findings highlight the gap between positive perceptions of vaccine safety and actual uptake rates, suggesting that additional factors, such as accessibility, misconceptions, or external influences, may play a role in vaccination behavior.

**Table 3:** Respondents’ attitudes and practices toward seasonal influenza vaccination.

Attitudes toward seasonal influenza vaccination	Category	*n* (%)
How confident are you in the safety of seasonal influenza vaccines?	Very confident	139 (31.1)
	Somewhat confident	108 (24.2)
	Neutral	129 (28.9)
	Somewhat concerned	50 (11.2)
	Very concerned	21 (4.7)
Vaccination practices		
Have you ever received a seasonal influenza vaccine?	No	156 (34.9)
	Yes	291 (65.1)
How often do you usually get the seasonal influenza vaccine?	Occasionally	87 (19.5)
	Every year	84 (18.8)
	No specific pattern	55 (12.3)
	Rarely	65 (14.5)
Did you receive the influenza vaccine this season?	No	263 (58.8)
	Yes	184 (41.2)

### Confidence and experiences with seasonal influenza vaccine effectiveness

Table 4 shows that while 59.5% of respondents are confident in the effectiveness of the seasonal influenza vaccine, 41.2% reported breakthrough infections. Among those, 27.2% gained confidence due to the rarity of such cases, 19.6% slightly lost confidence, 7.1% significantly lost confidence, and 46.2% remained unaffected.

**Table 4:** Respondents’ responses on confidence and experiences with seasonal influenza vaccine effectiveness.

Question	Category	*n* (%)
How confident are you in the effectiveness of the seasonal influenza vaccine in preventing illness?	Very confident	127 (28.4)
	Somewhat confident	139 (31.1)
	Neutral	113 (25.3)
	Somewhat unconvinced	48 (10.7)
	Not convinced at all	20 (4.5)
Have you or anyone you know ever experienced a breakthrough influenza infection despite receiving the seasonal influenza vaccine? *(Breakthrough infections occur when vaccinated individuals get the illness)*	No	263 (58.8)
	Yes	184 (41.2)
If yes, how has this experience affected your view of the seasonal influenza vaccine?	It increased my confidence in the vaccine due to the rarity of breakthrough infections	50 (27.2)
	Reduced my confidence in the vaccine slightly	36 (19.6)
	This significantly reduced my confidence in the vaccine	13 (7.1)
	It did not affect my view, as I understand no vaccine is 100% effective	85 (46.2)

The accessibility and convenience of seasonal influenza vaccination services in the community vary among respondents. A majority, 258 (57.7%), find the services easy to access. In contrast, 4 (0.9%) respondents consider them very difficult to access, and 1 (0.2%) is unaware of the method to access these services. Additionally, 47 (10.5%) respondents feel neutral about the accessibility, while 21 (4.7%) find it somewhat difficult, and 116 (26%) believe the services are slightly accessible.

### Factors influencing seasonal influenza vaccination

Table 5 shows a multifaceted influence on attitudes toward the seasonal influenza vaccine. Social pressure from peers is varied, with the highest proportion of respondents (30%) feeling neutral, 17.4% experiencing strong encouragement, and 14.5% encountering strong discouragement. Trust in health institution-provided information is significant, with 36.9% expressing complete trust and 32% moderate trust. Awareness regarding the vaccine’s importance is high, with 36.7% being very aware and 33.6% somewhat aware. The COVID-19 pandemic has notably impacted vaccination intentions, with 36.7% of respondents reporting an increased desire to vaccinate due to exposure concerns, while 36.2% indicated their decision was not significantly affected.

**Table 5:** Factors influencing seasonal influenza vaccination.

Question	Category	*n* (%)
To what extent do you feel social pressure or encouragement from friends, family, and colleagues to get the seasonal influenza vaccine?	Strong encouragement	78 (17.4)
	Moderate encouragement	106 (23.7)
	Neutral	134 (30)
	Moderate discouragement	64 (14.3)
	Strong discouragement	65 (14.5)
How much trust do you have in the information provided by health institutions regarding the seasonal influenza vaccine?	Complete trust	165 (36.9)
	Moderate trust	143 (32)
	Neutral	79 (17.7)
	Limited trust	43 (9.6)
	Distrust	17 (3.8)
How do you rate your general awareness of the importance of seasonal influenza vaccination in preventing the spread of influenza?	Very aware	164 (36.7)
	Somewhat aware	150 (33.6)
	Neutral	89 (19.9)
	Somewhat unaware	31 (6.9)
	Completely unaware	13 (2.9)
How has the COVID-19 pandemic affected your decision to receive the seasonal influenza vaccine?	Increased my desire to get vaccinated due to concern about exposure	164 (36.7)
	It did not significantly affect my decision	162 (36.2)
	Reduced my desire because I am already taking precautions	117 (26.2)
	Other	4 (0.9)

### Likelihood of receiving the seasonal influenza vaccine next season

**Figure 1:**
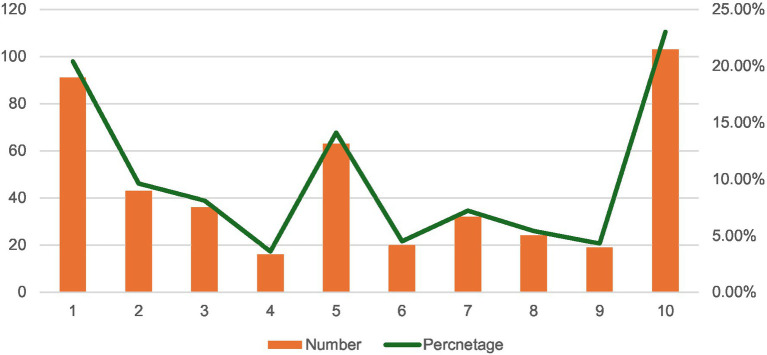
Likelihood of receiving the seasonal influenza vaccine next season.

The chart below illustrates the respondents’ intentions on a scale from 1 to 10. The data reveals a significant inclination toward both extremes, with 20.4% of respondents being very unlikely (1) and 23% being very likely (10) to get the vaccine. Intermediate levels show varying degrees of uncertainty or moderate likelihood, with notable peaks at 5 (14.1%) and lower percentages for other values. This distribution highlights an attitude toward vaccination, with a considerable portion of the population either strongly favoring or against receiving the influenza vaccine next season (Figure 1).

Table 6 demonstrates significant positive correlations between the likelihood of receiving the flu vaccine and factors such as trust in safety, trust in efficacy, peer pressure; trust in information, and awareness, with all associated *p-*values indicating statistical significance (*p* < 0.001). Conversely, accessibility did not exhibit a statistically significant relationship with vaccine uptake. *p-*value for the likelihood of receiving the flu vaccine is “N/A” because it represents the dependent variable, which inherently correlates perfectly (*r* = 1.0) with itself, rendering a significance test inapplicable. These results underscore the importance of trust and awareness as key influencing factors associated with flu vaccine uptake.

**Table 6:** Correlation of flu vaccine likelihood (*N* = 447).

Factor	Pearson correlation (*r*)	Significance (*p-*value)
Trust in safety	0.37	<0.001
Trust in efficacy	0.42	<0.001
Accessibility	0.054	0.251
Peer pressure	0.251	<0.001
Trust in information	0.341	<0.001
Awareness	0.372	<0.001
Likelihood of receiving flu vaccine	1.0	N/A

## Discussion

### Summary

The primary findings from this cross-sectional study reveal significant insights into the public perception and uptake of the seasonal influenza vaccine in Makkah region of Saudi Arabia. The study highlights a notable male dominance among respondents, with the majority being young adults. Despite a generally positive attitude toward the influenza vaccine, a significant portion of respondents did not receive the vaccine this season, indicating potential barriers to vaccine uptake. These barriers may include misconceptions about vaccine effectiveness and concerns about breakthrough infections, which many participants reported. The study underscores the importance of addressing these barriers through targeted public health interventions and educational campaigns. Furthermore, the study identifies key factors influencing vaccination decisions, such as social pressure, trust in health institutions, and concerns related to the COVID-19 pandemic. Social influence from peers and family, along with trust in the information provided by health institutions, plays a crucial role in shaping individuals’ attitudes toward vaccination. The heightened awareness and increased desire to vaccinate due to COVID-19 concerns present an opportunity to leverage this awareness for promoting influenza vaccination. These findings suggest that enhancing trust and leveraging social networks can significantly improve vaccination rates. Public health strategies should focus on clear communication about vaccine safety and efficacy and improving the accessibility of vaccination services to address the population’s diverse needs in Makkah region.

### Main discussion

The findings from this cross-sectional study provide a comprehensive understanding of the demographic characteristics and attitudes toward influenza vaccination in Makkah region. The age distribution, with a significant proportion of respondents aged 18–30 years (44.5%), suggests that younger adults are more engaged in health questionnaires, possibly due to higher levels of education or greater access to digital platforms. Educational attainment plays a crucial role in health behavior, and the high percentage of respondents holding a bachelor’s degree (57.5%) indicates that educated individuals may be more aware of the benefits of vaccination. However, the finding that 58.8% of respondents did not receive the influenza vaccine this season despite a generally positive attitude toward its safety and effectiveness highlights the presence of barriers to vaccination that go beyond education and awareness. One of the key insights from this study is the moderate level of confidence in the vaccine’s effectiveness (59.5%) and the significant number of breakthrough infections reported (41.2%). This suggests that while there is general trust in the vaccine, experiences of breakthrough infections may undermine confidence. An important consideration is that these infections are often self-diagnosed, making it difficult to prove their validity, as most people need help distinguishing between the common cold and influenza. Public health campaigns need to address these concerns by providing precise and accurate information about the expected efficacy of the vaccine and the normalcy of breakthrough infections due to the virus’s mutability. Social pressure and trust in health institutions emerged as significant factors influencing vaccination decisions. This underscores the importance of community and familial influence on health behaviors. Public health initiatives should consider involving community leaders and trusted figures to advocate for vaccination, thereby leveraging social networks to improve vaccine uptake. Trust in health institutions (with 36.9% expressing complete trust) indicates that efforts to enhance transparency and communication from these bodies can positively influence vaccination rates. Another critical finding is the influence of the COVID-19 pandemic on vaccination decisions. The increased desire to vaccinate due to COVID-19 (36.7%) indicates that the pandemic has raised awareness about the importance of vaccination in preventing respiratory illnesses. This raised awareness can be harnessed to promote influenza vaccination and other routine immunizations that may have been neglected during the pandemic. This result is aligned with a systematic review published in 2022, which reported that the COVID-19 pandemic has significantly boosted the intention to vaccinate against influenza worldwide. Analysis of 27 studies with 39,193 participants revealed a 50% increase in vaccination intention for the 2020/21 season compared to pre-COVID-19 rates. This trend was consistent across age, gender, and occupation. Key factors driving this increase include historical vaccine acceptance and perceptions of influenza severity and vaccine safety. The pandemic presents a unique opportunity to promote influenza vaccination and reduce vaccine hesitancy (18). A study among healthcare workers in Saudi Arabia found that those who perceived a greater negative impact from the COVID-19 pandemic were 40% more likely to receive the influenza vaccine (19). Additionally, research in Jeddah, Saudi Arabia, indicated that the COVID-19 pandemic had little effect on individuals’ decisions to receive the influenza vaccine, suggesting that other factors may play a more significant role in vaccination uptake (20). Accessibility to vaccination services was generally rated as easy (57.7%), yet a significant portion of the population did not find it easy to access these services. This indicates a need for more widespread and convenient vaccination locations and enhanced communication about where and how to get vaccinated. This study’s findings on influenza vaccine uptake and hesitancy align with trends in Saudi Arabia and the broader Middle East. Prior research in Riyadh has reported similar vaccination rates and hesitancy levels, influenced by misconceptions about vaccine safety, perceived effectiveness, and social influence (21). Studies in other Gulf countries, including the UAE, Kuwait, and Oman, have reported varying vaccination rates among healthcare workers, with 24.7% in the UAE, 67.2% in Kuwait, and 46.4% in Oman. The primary motivator for vaccination was self-protection (59%), while the most common barrier was a lack of time (31.8%). Other factors influencing vaccine hesitancy included unawareness of vaccine availability (29.4%), vaccine unavailability (25.4%), doubts about efficacy (24.9%), lack of information about importance (20.1%), and concerns about side effects (17.3%) (22). Educational attainment is crucial in health behavior, particularly regarding vaccine acceptance. A 2013 study in Bangkok found that health education significantly increased influenza vaccine acceptance among older adults, particularly those with lower education levels and no prior vaccination history. After targeted educational interventions, acceptance rates rose from 83.3 to 92.6% (23). However, this trend was not observed in our study, likely due to Makkah’s unique demographic and cultural context, where a substantial proportion of the population is already aware of the potential spread of influenza during Hajj and Umrah. While the Bangkok study was referenced due to its structured health education intervention, it is essential to acknowledge that more regionally relevant studies should also be compared. The findings in Makkah emphasize the importance of addressing vaccine misconceptions and enhancing public trust. Given the region’s large influx of visitors during Hajj and Umrah, targeted vaccination campaigns focusing on high-risk populations and leveraging healthcare professionals as trusted sources of information may improve uptake. Comparative analysis with global trends underscores the need for more structured awareness programs, integrating lessons from countries with successful vaccination strategies (13). Given the region’s large influx of visitors during Hajj (pilgrim) and Umrah, Makkah presents a unique context where high population density and international travel increase the risk of influenza transmission. This reinforces the importance of vaccination campaigns in reducing disease burden in such an environment. The unique setting of Makkah requires targeted public health strategies that prioritize accessibility, education, and vaccine advocacy, particularly for high-risk populations such as older adult individuals, those with chronic illnesses, and healthcare workers. Trust in vaccine safety and efficacy emerged as key influencing factors, suggesting that individuals who perceive the vaccine as safe and effective are more likely to receive it. Similarly, the significant associations with peer pressure and trust in information indicate that social influences and reliable communication sources play pivotal roles in shaping vaccination decisions. Awareness was also significantly correlated, highlighting the importance of educational campaigns in improving vaccine uptake. In contrast, accessibility did not show a significant relationship, potentially reflecting that structural barriers to vaccine access may be less pronounced in the studied population or that trust, and awareness exert stronger influences. These findings collectively underscore the need for targeted interventions focusing on building trust, enhancing awareness, and leveraging social influences to improve flu vaccination rates. Overall, the study highlights several areas for intervention to improve influenza vaccination rates in Makkah region. Efforts should focus on addressing misconceptions about vaccine effectiveness, leveraging social and community influences, enhancing trust in health institutions, and improving the accessibility of vaccination services. By addressing these factors and considering the unique dynamics of Makkah region, public health initiatives can more effectively increase vaccination uptake and thereby reduce the prevalence of influenza and its associated complications in Makkah region.

### Limitations and strengths

One limitation of the study is the use of a snowballing sampling technique, which may introduce selection bias and limit the generalizability of the findings. Additionally, the reliance on self-reported data could result in response bias. The study’s cross-sectional nature limits the ability to establish causality between the identified factors and vaccination uptake. Moreover, the study did not account for potential confounders such as socioeconomic status, underlying health conditions, and previous vaccination history, which could influence vaccination behavior. The sample size of 450 respondents, while sufficient for meaningful statistical analysis, is relatively smaller compared to other studies conducted on similar topics in Saudi Arabia, where sample sizes often about 1,000 participants (24). While the sample size may limit broader generalizability, it still provides valuable insights into vaccine hesitancy and uptake trends within Makkah region. Future research with a more prominent and representative sample would help strengthen the findings and allow for greater external validity. Despite these limitations, the study comprehensively analyses multiple factors influencing vaccination decisions, offering valuable insights for targeted interventions. Additionally, the focus on Makkah region, with its unique demographic and cultural characteristics, adds a significant contextual understanding to the factors influencing vaccination uptake in this area with a high visitor population.

## Conclusion

This study contributes significantly to understanding the factors associated with influenza vaccine uptake in Makkah region. The findings highlight the need for targeted public health initiatives to address barriers to vaccination and reinforce positive attitudes toward the influenza vaccine. In practice, these insights can inform the development of tailored public health campaigns that address specific concerns and leverage social influences and trust in health institutions. For research, this study provides a foundation for further exploration into the factors affecting vaccine uptake in different demographic and geographic contexts. Future research could investigate interventions to improve vaccine confidence and uptake, mainly focusing on addressing breakthrough infections and enhancing communication strategies about vaccine effectiveness.

## Data Availability

The raw data supporting the conclusions of this article will be made available by the authors, without undue reservation.
